# Primary Care Patients’ Preferences and Willingness to Pay for Home-Based Healthcare in China

**DOI:** 10.1093/geroni/igaf122.3168

**Published:** 2025-12-31

**Authors:** Sixian Du, Yaqing Liu

**Affiliations:** Huazhong University of Science and Technology, Wuhan, Hubei, China (People’s Republic); Huazhong University of Science and Technology, Hubei, Hubei, China (People’s Republic)

## Abstract

Population aging and the increasing burden of chronic diseases have created urgent demand for high-quality, patient-centered home-based healthcare and support services (HHSS) for older adults. However, evidence on patient preferences for HHSS in developing countries remains limited. This study aimed to identify preferences and willingness to pay (WTP) for HHSS among primary care patients in China, focusing on the aging population. A discrete choice experiment (DCE) was conducted with 312 patients from 13 community health centers in Wuhan and Kunming between January and May 2023. Five attributes were included: scope of services, type of healthcare professional, service institution, insurance reimbursement, and visiting fee. Mixed logit models revealed that the scope of services was the most influential attribute (relative importance 67.33%), with strong preferences for physician-led diagnostic and treatment services over preventive care, mental health support, or daily living assistance. Participants favored services provided by hospitals (19.84% importance) and qualified physicians (12.42% importance), reflecting trust in formal medical institutions. Latent class analysis identified three distinct subgroups: 60.2% prioritized comprehensive medical services; 16.6% preferred services delivered by reputable institutions and professionals; and 23.2% were highly sensitive to cost and reimbursement. WTP analysis indicated patients were willing to pay more for diagnostic and treatment services but less for mental health or care support services. These findings suggest that expanding physician-led, home-based medical care while addressing affordability barriers is critical for advancing geriatric HHSS in China. Policymakers should prioritize developing integrated, multidisciplinary HHSS models to promote healthy aging and support aging in place.

